# Two-dimensional airflow modeling underpredicts the wind velocity over dunes

**DOI:** 10.1038/srep16572

**Published:** 2015-11-17

**Authors:** Britt Michelsen, Severin Strobl, Eric J. R. Parteli, Thorsten Pöschel

**Affiliations:** 1 Friedrich-Alexander-Universität Erlangen-Nürnberg, Erlangen, Germany; 2 Department of Geosciences, University of Cologne, Germany

## Abstract

We investigate the average turbulent wind field over a barchan dune by means of Computational Fluid Dynamics. We find that the fractional speed-up ratio of the wind velocity over the three-dimensional barchan shape differs from the one obtained from two-dimensional calculations of the airflow over the longitudinal cut along the dune’s symmetry axis — that is, over the equivalent transverse dune of same size. This finding suggests that the modeling of the airflow over the central slice of barchan dunes is insufficient for the purpose of the quantitative description of barchan dune dynamics as three-dimensional flow effects cannot be neglected.

Sand dunes occur in many deserts and coastal areas. They form when the wind is strong enough to transport grains in saltation — which consists of particles moving in nearly ballistic trajectories and ejecting new grains upon collision with the bed^1^. The quantitative understanding of dune dynamics is a relevant problem for the society in particular as dune mobility is largely responsible for the spread of desertification.

One of the dune types with highest mobility is the barchan (Fig. 1). Barchans move on top of bedrock in areas where the wind blows nearly steadily from the same direction. They have a crescent shape with two limbs pointing in the migration trend and can cover a distance of 30–100 m in a year. The morphology and dynamics of barchans have been the subject of intense field investigation since the pioneering works by Bagnold^1^,^2^,^3^,^4^,^5^,^6^. However, in order to predict the dynamics of barchan dunes, a quantitative understanding of the turbulent wind field over the dune profile, which dictates the sand flux and the rates of erosion and deposition on the dune topograhy, is required. As a matter of fact, the migration velocity of the dune scales with the sand flux *Q*, which is the main quantity characterizing dune dynamics^1^. However, *Q* is a non-linear, increasing function of the wind shear velocity u_*_, which is proportional to the mean flow velocity gradient in turbulent boundary layer flow^7^,^8^,^9^. Specifically, *Q* scales approximately with 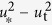 when the wind is close to the minimal threshold wind shear velocity for sand transport, *u*_t_, and with 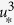 for large values of u_*_^1^,^9^,^10^. Therefore, an erroneous computation of the wind speed-up over the dune leads to erroneous predictions of the sand flux profile. And since the modeling of sand transport is the main factor affecting the calculation of dune dynamics, the accurate description of the wind velocity profile over the dune is an essential prerequisite for correctly predicting dune morphodynamics, in particular the migration velocity and the shape evolution of the dune.

Measurements of the wind velocity over barchan dunes have been performed by several authors^11^,^12^,^13^,^14^. For instance, Wiggs *et al.*^12^ reported measurements of the wind velocity on the windward side of a barchan dune in Oman. The measurements by these authors revealed essential features of the wind velocity profile over barchans which must be reproduced by a reliable numerical tool for the simulation of dune dynamics. Indeed, numerical modeling is indispensable to achieve a detailed characterisation of the turbulent wind flow over large-scale spatial extents, since the resolution required for the quantitative understanding of the long-term topography evolution cannot be matched from point measurements as they are performed in field investigations. In the present work, we use Computational Fluid Dynamic modeling in order to calculate the average turbulent wind field over a barchan dune. We will show that our simulations reproduce the behavior of the wind velocity profile over barchans observed in the field, including the flow deceleration at the upwind dune’s toe, the speed-up of flow velocity toward the crest and the separation bubble in the dune lee.

By means of Computational Fluid Dynamic simulations, we solve the averaged Navier-Stokes equations for the turbulent wind field over the terrain comprising the flat ground and the barchan dune. In the absence of the dune, the wind velocity *U* increases logarithmically with the height *z* above the ground, that is *U*(*z*) = [u_*_/*κ*] ⋅ *ln*(*z*/*z*_0_), where u_*_ is the wind shear velocity, *κ* = 0.4 is the von Kármán constant and *z*_0_ is the surface roughness^7^,^8^. The near-surface shear stress is constant and given by 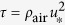, with *ρ*_air_ = 1.225 kg/m^3^ standing for air density. However, the dune introduces a barrier to the wind and modifies the shear stress profile along the terrain. The wind velocity (*U*) measured at a given height above the surface thus changes along the terrain’s longitudinal profile in the presence of the dune. Since this velocity is an important parameter considered in field investigations, in our simulations we investigate the evolution of *U* with the along-wind position in the presence of the barchan dune.

In our simulations, a barchan dune is placed in the center of a numerical wind-tunnel of height *H*_ch_ = 30 m and along-wind width of *L*_ch_ = 200 m. Figure 2a shows the height profile of the longitudinal cut along the symmetry axis of the simulated barchan — that is, the central slice of the barchan (see Section Methods). This profile corresponds to the measured profile of a barchan dune in Oman, investigated by Wiggs *et al.*^12^. The height of the dune is 10 m and the distance from the windward toe to the foot of the dune at the lee is about 90 m. Moreover, the slip face at the lee makes an angle of 34° with the horizontal, which is the angle of repose of sand.

We perform two types of numerical simulations. In the first one we compute the wind flow over the two-dimensional profile shown in Fig. 2a, while in the second type we consider the full three-dimensional dune profile in Fig. 2b, which has been constructed from the two-dimensional profile Fig. 2a as described in the Section Methods.

The near surface wind flow is computed using the open-source CFD software package OpenFOAM^15^. In order to solve the wind flow, we apply a Reynolds-Averaged Navier-Stokes (RANS) solver based on the PIMPLE algorithm^16^, whereas turbulence is modeled using the Re-Normalisation Group (RNG) *k* − *ε* model^17^,^18^,^19^,^20^,^21^. As a matter of fact, the mixing length model for turbulence cannot be applied when there is flow separation or recirculation^7^. Moreover, it was shown that the standard *k* − *ε* model performs poorly for problems where the surface has sharp discontinuities in its first derivative, such as in the case of a dune with a slip face^22^. However, the RNG *k* − *ε* model has proven to describe the main characteristics of turbulent flows in such problems with quantitative agreement with measurements — for instance the size of the separation zone of the flow over a backstep where the lee side makes an angle of 90° with the horizontal (see ref. 22 for a discussion). This model, outlined elsewhere^23^,^24^, describes the transport of turbulent kinetic energy (*k*) and the turbulent dissipation (*ε*), which determines the scale of the turbulence. These variables are determined using the equations,


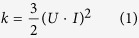


and


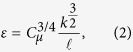


where *C*_*μ*_ is a turbulence model constant, 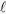 is the turbulence length-scale, and *I* stands for the turbulence intensity. In our simulations, we take *C*_*μ*_ = 0.09 and *I* = 0.01. Furthermore, the turbulent length scale 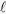 is used to estimate the characteristics of turbulence at the inlet of the CFD simulation. This length-scale should be smaller than the characteristic length-scale of the system, as otherwise turbulent eddies larger than the system’s size would be considered. Typically a value of 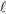 around 7% of the typical length-scale of the system is used^15^,^25^. Therefore, here we take 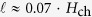, where *H*_ch_ is the height of the channel. We note that, although it would be interesting to investigate the sensitivity of the outcomes to these model parameters, the aforementioned values have been chosen here as they were adopted in previous modeling of boundary layer flow over different types of dune topography thereby leading to good agreement with measurements^19^,^21^,^26^. Moreover, we take *U* = 8 m/s, which is larger than the minimal threshold for sand transport (about 5 m/s at a height of 1 m^1^) and is consistent with realistic wind velocity values in barchan dune fields^13^.

## Results

**Figure 2:**
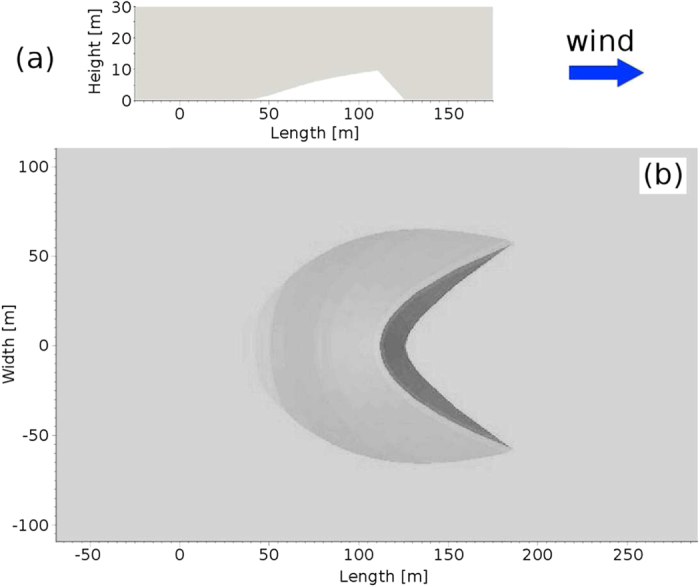
Two-dimensional dune profile (a) and three-dimensional barchan dune shape (b) considered in the simulations.

**Figure 3:**
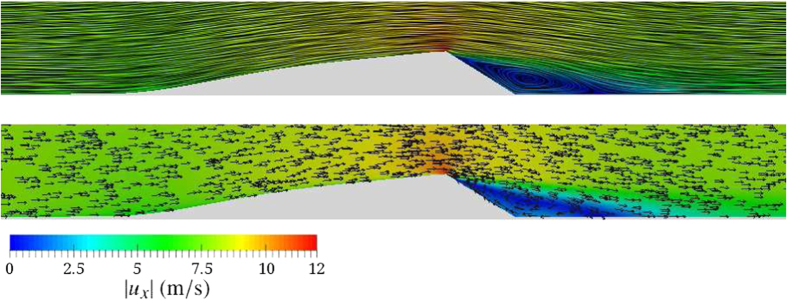
Streamlines (top) and velocity vectors (bottom) of the wind velocity field over the two-dimensional dune profile in Fig. 2a. Wind blows from left to right.

Figure 3 shows the streamlines (top) and the velocity vectors (bottom) of the wind velocity field over the transverse dune in Fig. 2a obtained from our calculations. As we can see from Fig. 3, there is a zone of recirculating flow at the lee side of the dune which is characteristic of the flow separation phenomenon that takes place at the sharp brink. The development of this zone, also known as separation bubble, has been reported in numerous field studies, experiments and numerical simulations^17^,^27^,^28^,^29^. Inside the separation bubble, net transport in the downwind direction nearly vanishes. However, depending on the wind velocity, recirculating transport in the dune lee can occur and even contribute to shape the slip face of the dune^20^. The separation bubble plays an important role for the dune shape as the dune wake serves as a sand trap, which prevents sand deposited from avalanches along the slip face to be blown away from the dune.

The results of our simulations show that the velocity magnitude at the windward side of the dune varies with the distance downwind. Close to the upwind foot of the dune, the wind velocity is reduced, thereafter increasing again along the windward side towards the dune crest. Moreover, the position of the maximum of the wind velocity is approximately at the highest position of the dune, that is the crest position, which in the case of the dune of Fig. 3 coincides with the position of the brink. This behavior is consistent with field observations of ref. 13, who measured the wind profile over a barchan with sharp brink and also found that the maximum of wind velocity is at the dune brink.

**Figure 4:**
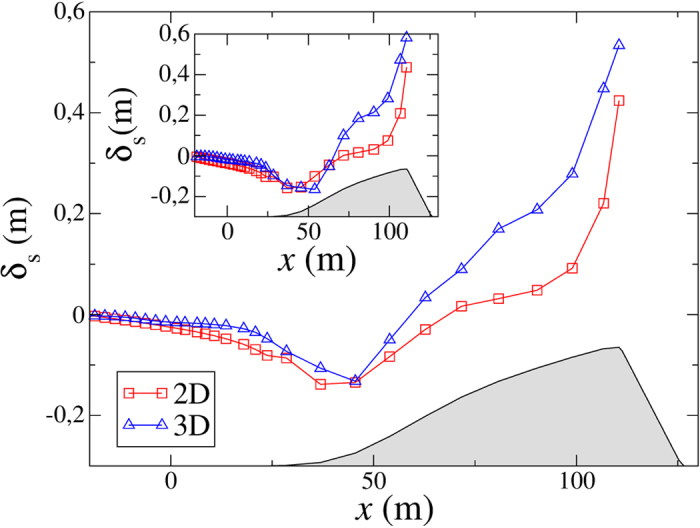
Main plot: Fractional speed-up ratio of the wind velocity over the dune topography, computed with Eq. (3), for a height of 1 m above the surface. The results obtained for the two-dimensional dune profile in Fig. 2a are denoted in the squares. Moreover, the results from the three-dimensional calculations are denoted by the triangles, which correspond to the curve *δ*_*s*_ over the longitudinal cut along the symmetry axis of the three-dimensional barchan shape depicted in Fig. 2b. The inset shows the corresponding values of *δ*_*s*_ obtained at a height of 60 cm above the surface.

In order to quantify the effect of the dune profile on the wind velocity, we compute the fractional speed-up ratio *δ*_*s*_ of the wind velocity at a given height above the surface^30^,


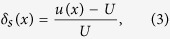


where *u*(*x*) is the wind velocity at downwind position *x* and *U* is the wind velocity over the flat ground (in the absence of the dune). In the main plot of Fig. 4, the squares denote *δ*_*s*_(*x*) for a height of 1 m above the surface, as obtained from the calculations over the two-dimensional dune profile (Fig. 2a). Furthermore, the squares in the inset of Fig. 4 denote *δ*_*s*_(*x*) obtained for a height of 60 cm above the surface, using the same dune profile.

Next we compute the wind flow over the three-dimensional dune profile of Fig. 2b. Again, we calculate the fractional speed-up ratio of the wind velocity, *δ*_*s*_, over the longitudinal cut along the symmetry axis of the barchan. The results of this calculation for two different heights above the surface are shown in Fig. 4, together with the corresponding results from the two-dimensional calculations. Specifically, the triangles in the main plot and inset of this figure denote the calculations corresponding to a height of 1 m and 60 cm, respectively.

As we can see from Fig. 4, the same features of *δ*_*s*_ observed in the field and in the two-dimensional calculation are observed in the simulation using the three-dimensional barchan shape. However, quantitatively the results obtained with the 2d and 3d dune shapes differ. In particular, the increase of *δ*_*s*_(*x*) with *x* from the windward foot toward the crest is faster in the simulation using the three-dimensional dune shape than in the calculation of the airflow over the two-dimensional dune. This difference is an important result in particular since it is well-known that the shape of the central slice of a barchan can be well approximated by a transverse dune — which has a nearly fixed profile in the direction orthogonal to the wind^31^,^32^. Indeed, the three-dimensional barchan shape investigated here was modeled from the transverse dune in Fig. 2a. But even though the central slice of the barchan has exactly the same shape as the two-dimensional transverse dune, the airflow over these dunes behaves differently. This difference may be understood by noting that the three-dimensional shape of barchans and transverse dunes are fundamentally different. While the horizontal profile of a transverse dune in the direction perpendicular to the wind is straight (invariant), the one of the barchan is nearly parabolic^5^. Thus, we expect differences in the airflow behavior over these two different three-dimensional geometries.

Moreover, it is also well-known that the flow profile on the windward side of barchans and transverse dunes is affected by the flow structure in the lee side. In particular, one important difference between the flow within the separation bubble of barchans and transverse dunes is the occurrence of large three-dimensional flow patterns (with horizontal cross-wind components) arising in the lee of the former dunes. Using large-eddy simulations, Omidyeganeh *et al.*^33^ showed that two counter-rotating streamwise vortices develop along each of the limbs of a barchan. These authors also showed that these vortices direct high-momentum fluid toward the centerline symmetry plane of the barchan, while low-momentum fluid is directed near the bed away from this centerline. Mean secondary flow is introduced by the three-dimensional barchan shape across the whole topography thereby substantially altering the characteristics of the turbulence on the windward side of the dune^33^. Here we have shown that the wind velocity increases faster along the windward side of the longitudinal cut along the symmetry axis of a barchan than it does on the stoss side of a transverse dune of same shape. This faster increase can be understood by noting that the three-dimensional, counter-rotating vortices in the separation bubble of the barchan dune — which develop along the dune limbs as shown in ref. 33 — lead to a larger resistance to the downward deflection of the streamlines associated with the upper flow above the separation bubble compared to the two-dimensional dune. Indeed, this deflection is caused by a negative pressure perturbation at the crest, which keeps the streamlines of the flow over the dune attached to the surface^34^,^35^. The increased resistance of the flow in the separation bubble to this pressure perturbation leads to a higher compression of the streamlines over the 3D dune surface than over the 2D one. Therefore, the flow streamlines get closer to each other over the 3D dune, thus leading to a higher basal shear stress compared to the 2D dune. However, qualitatively the behavior of the wind velocity profile is the same for both dune types as shown in Fig. 4.

We remark that the wind velocity profile depends on the height profile of the dune, which can be very different depending not only on wind conditions but also on dune size. In our simulations we have taken a dune profile in which the crest position (that is, the position of maximal height) coincides with the position at which the sharp brink separating windward and lee sides occurs. Although such a profile is representative for large dunes, it was shown that as the barchan size decreases toward the minimal size (which corresponds to a height of about 50 cm^1^,^31^), the dune shape becomes rounded with brink and crest separated by a distance that is negatively correlated with the dune size^5^. Previous authors showed that the separation bubble of topographic structures mimicking the shape of rounded transverse dunes is smaller than the zone of recirculating flow of sharp-crested dunes of same size^36^. Since the turbulence characteristics depend on the dune shape, and since the dune shape depends on the dune size, the behavior of the wind profile on the windward side depends on the dune size. Future work is thus necessary for investigating how the results obtained in the present work change with the dune size.

## Discussion

We investigated the airflow over a barchan dune under constant wind direction using Computational Fluid Dynamic modeling. Our simulations reproduce essential features of this airflow which have been reported from field investigations, in particular the formation of a zone of recirculating flow in the dune lee, the reduction of the wind velocity at the dune upwind foot and the increase in flow speed over the windward side toward the crest. Moreover, we found that this increase is stronger in the simulations considering a full three-dimensional dune shape than in the two-dimensional modeling using the longitudinal cut along the symmetry axis of the dune.

It is well-known that the *shape* of the central slice of a barchan can be, indeed, well approximated by the profile of a transverse dune of same dimensions. However, what we have shown here is that flow effects occurring over the three-dimensional barchan shape may lead to very different wind velocity profiles compared to the one obtained for a two-dimensional dune, *under the same wind speed and considering the same dune size*. Therefore, our simulations show that three-dimensional effects cannot be neglected in the calculation of the airflow over barchan dunes.

It should be further noted that real barchans may have a broad range of three-dimensional shapes, depending on several conditions including grain size and interdune flux — see for example refs 1,37. Correspondingly, the size of the wake region in the lee, the characteristics of the three-dimensional flow effects over the topography and, thus, the deviations in wind velocity profile from the predictions obtained with the two-dimensional calculations may vary considerably over the different barchan shapes occurring in Nature. Therefore, a systematic investigation using barchan dune models of different shapes and sizes, in particular of different values of lateral width and aspect ratio (height over longitudinal length) as well as curvature of the dune crest, is required in order to further improve our understanding of the wind field over dunes. For example, ref. 36 fitted the windward side of transverse dunes using half circles in order to obtain various rounded and sharp-crested bedforms by changing the crest to brink distance, while ref. 17 modeled triangular dune shapes with different values of aspect ratio. However, such a systematic investigation is out of the scope of our study. Instead, the present manuscript focuses on the modeling of the wind flow over one real dune profile, which has been taken from the field data by ref. 12. Furthermore, we note that the differences in speed-up obtained for the 3D and 2D dunes should also depend on the model used to obtain the three-dimensional shape from the real two-dimensional profile. However, ref. 33 showed that the emergence of the counter-rotating vortices in the separation bubble of the three-dimensional barchan shape leads to an increase in the high-momentum fluid toward the centerline symmetry plane of the barchan. *Qualitatively*, the conclusion that a higher speed-up of the wind velocity occurs for the three-dimensional barchan shape (compared to the equivalent two-dimensional transverse dune) should be, thus, robust with respect to the three-dimensional barchan model used. Based on the results presented here, it would be interesting to perform systematic investigations in order to improve the quantitative assessment of the wind flow over different barchan morphologies, and to quantify the discrepancies between predictions of wind speed-up obtained from the full three-dimensional model and using the equivalent transverse dune. Furthermore, the implications of our results for dune dynamics should be also investigated by coupling the wind model with a sand transport model^10^,^32^,^38^,^39^,^40^,^41^,^42^.

It is interesting to remark that, for the sharp-crested dune profile investigated here, the maximum of the wind velocity occurs at the crest position, which coincides with the position of the sharp brink as can be seen in Fig. 3. In contrast, the wind profile over smooth hills or domes without slip face has a different behavior. For such bedforms, the maximum of the wind velocity is shifted *upwind* of the dune crest as shown in field measurements^43^. This phase advance of the maximum wind velocity relative to the hill’s crest is due to the inertia of the turbulent wind velocity fluctuations, which counteracts the deflation of the flow streamlines over the surface — upwards on the windward side and downwards on the downwind side^31^. The analytical model for the average turbulent surface shear stress over smooth terrains developed by Jackson and Hunt^30^ reproduces this phase advance. Indeed, this analytical model can be applied only for rounded-crested domes or low hills that do not have slip face, since it is not valid when flow separation occurs. In order to obtain the surface shear stress over the windward side of a transverse dune using an analytical model, ref. 31 employed the ansatz proposed by Zeman and Jensen^44^, which consists of applying the model of Jackson and Hunt^30^ to compute the shear stress over the *envelope* comprising the dune windward side and the separation streamline of the flow in the dune lee (that is, the streamline that separates the upper flow from the separation bubble in the dune lee). In this ansatz, the wind flow inside the separation bubble is then set as equal to zero, since net transport inside the bubble nearly vanishes^35^. With respect to the envelope defined by the dune windward side and the separation streamline, the position of maximal wind velocity obtained from the analytical model is, indeed, shifted *upwind* of the envelope’s crest (see refs 9,31,32,35). However, with respect to the dune profile, the maximum occurs at the brink of the dune, which is in consistence with field observations^13^ and with our simulation results (Fig. 3).

The present study should be now continued with the investigation of the airflow over complex dune topographies which are ubiquitous in real dune fields. In particular, barchans often occur closely spaced forming barchanoidal chains^45^,^46^,^47^. Indeed, the proximity of the dunes in barchanoidal fields is known to strongly affect the wind profile over the topography. This profile should be now investigated in a systematic manner for different three-dimensional (realistic) barchanoidal shapes following preliminary works with two-dimensional arrays of transverse dunes^18^,^48^ and transverse ridges superposed with sinuous-crested dune shapes^49^.

Moreover, barchan dunes occurring in areas where the wind direction changes seasonally may develop an asymmetric shape with one elongated limb eventually evolving into a longitudinal seif dune^50^,^51^. In order to correctly model this process it is important to accurately describe the secondary flow effects in the lee of a barchan oriented obliquely to the prevailing wind. However, these effects are still uncertain and should be investigated by means of CFD simulations like the ones presented here. Such simulations should be further applied to investigate the airflow over other dune types, in particular star dunes and those exotic dune shapes occurring on Mars^32^.

## Methods

### Dune geometry and discretization scheme

To obtain the two-dimensional dune profile used in our calculations (Fig. 2a), Fig. 4 of ref. 12 has been digitized, whereupon several points along the height profile of the measured dune have been defined for the mesh generation (described below). Moreover, the three-dimensional profile is constructed from the two-dimensional profile in Fig. 2a using the open-source software SALOME^52^ and assuming a total along-wind width of 165 m and a cross-wind width of 130 m. To generate the barchan, the two-dimensional profile in Fig. 2a has been extruded in the cross-wind direction by using a three-dimensional envelope. This envelope has been obtained by compressing the surface of the upper half of a sphere, centered at the position of the dune crest and at height *z* = 0, such as to conform its central longitudinal slice to the height profile of the dune in Fig. 2a. Subsequently, the downwind half of the envelope has been removed and the longitudinal slices displaced downwind such as to obtain the dune shape in Fig. 2b.

In order to numerically compute the airflow, the mesh must be defined. For the two-dimensional simulation, the mesh is generated with the *blockMesh* utility supplied by OpenFOAM, and consists of hexahedral blocks. Edges of the blocks can be lines, arcs or splines. To refine the mesh a number of cells in each direction of the block can be adapted. We define a fine graded mesh which consists of 17,500 cells and has been refined at the lee of the dune, since in this area a zone of recirculating flow develops and due to the sharp brink of the dune a higher resolution is necessary at the lee as discussed e.g. in ref. 20. Moreover, in order to construct the three-dimensional barchan profile from the mesh created with *blockMesh*, 15 points from the dune windward side are sampled. The mesh is then created in SALOME using an automatic tetrahedral generator and is subsequently converted to an hexahedral mesh in OpenFOAM. Furthermore, the mesh near the surface is refined as in the two-dimensional simulation. The final mesh consists of 340,818 hexahedral and 9,361 polyhedral cells.

### Boundary conditions

The values of turbulent parameters *k* and *ε* at the inlet and top wall are computed using Eq. (1) and (2), respectively. At the outlet, the zero gradient boundary condition is applied, while at the bottom wall, that is at the surface comprising the flat ground and the dune, *k* = *ε* = 0. Furthermore, the pressure is assumed to have a constant value of 10^5^ Pa at the inlet as well as the top (which is the atmospheric pressure), while the zero gradient boundary condition is taken for the pressure at the bottom wall and at the outlet. Finally, a free stream velocity of 8 m/s is applied at the inlet and at the top boundary, while at the outlet zero gradient condition is applied for the velocity. At the bottom wall, no-slip boundary condition is applied for the velocity. The value of roughness *z*_0_ is about 100 *μ*m and the shear velocity upwind of the dune is about u_*_ ≈ 0.4m/s^20^. In the simulations, symmetry boundary conditions (that is, periodic boundary conditions applied to simulate an infinite domain) are applied to both vertical planar faces of the simulation box in the direction perpendicular to the wind.

## Additional Information

**How to cite this article**: Michelsen, B. *et al.* Two-dimensional airflow modeling underpredicts the wind velocity over dunes. *Sci. Rep.* **5**, 16572; doi: 10.1038/srep16572 (2015).

## Figures and Tables

**Figure 1:**
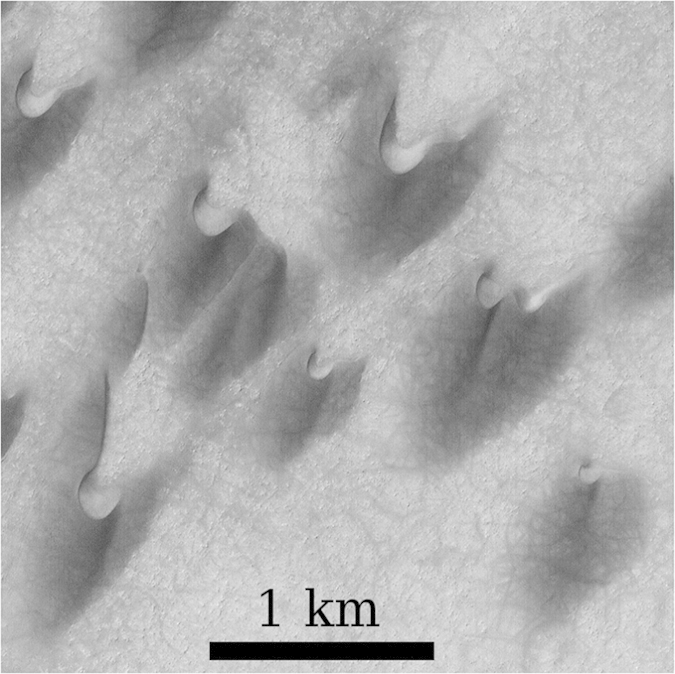
Barchan dunes in the Arkhangelsky crater on Mars, near 41°2S, 25°0W (image courtesy of NASA/JPL/MSSS).
